# Quantitative assessment of temporomandibular joint space in bruxers before and after occlusal splint therapy: A CBCT and MRI-based study

**DOI:** 10.6026/973206300211760

**Published:** 2025-06-30

**Authors:** Chirag R. Vaniya, Suvansh Gupta, Sourav Sen, Neha Bhadoria, Sadananda Hota, Sneha Sinha

**Affiliations:** 1 Department of Prosthodontics and Crown & Bridge, Government Dental College and Hospital, Jamnagar, Gujarat, India; 2 Department of Orthodontics and Dentofacial Orthopedics, Genesis Institute of Dental Sciences & Research, Ferozpur, Punjab, India; 3 Department of Public Health Dentistry, Maharishi Markandeshwar College of Dental Sciences and Research, Mullana, Ambala, Haryana, India; 4 Department of Pedodontics and Preventive Dentistry, Index Institute of Dental Sciences, Malawanchal University, Indore, Madhya Pradesh, India; 5 Department of Prosthodontics, Kalinga Institute of Dental Sciences, KIIT Deemed to be University, Bhubaneswar-751024, Odisha, India; 6 Consultant Prosthodontist and Implantologist, Pune, Maharashtra, India

**Keywords:** Bruxism, temporomandibular joint disorders, occlusal splints, magnetic resonance imaging (MRI)

## Abstract

Occlusal splint therapy is a widely utilized conservative intervention for managing bruxism-associated myofascial pain and temporomandibular joint (TMJ) dysfunction. A prospective observational study evaluated 112 patients using Cone Beam Computed Tomography (CBCT) and Magnetic Resonance Imaging (MRI) at baseline and after six months of nocturnal splint therapy to assess osseous and soft tissue TMJ changes. Thus, occlusal splint therapy effectively promotes joint space normalization and soft tissue recovery in bruxism patients with TMJ myofascial pain, irrespective of age and gender differences.

## Background:

Bruxism is a parafunctional activity characterized by involuntary grinding or clenching of the teeth, often occurring subconsciously during sleep or wakefulness. It is a multifactorial condition that has been significantly associated with temporomandibular joint (TMJ) disorders, including joint pain, disc displacement and condylar remodeling. Persistent biomechanical stress induced by bruxism may alter joint loading patterns, leading to degenerative or adaptive osseous changes in the TMJ [1]. Occlusal splint therapy-particularly stabilization splints-is a widely adopted conservative intervention aimed at redistributing occlusal forces, reducing muscular hyperactivity, and minimizing mechanical overload of the TMJ [2, 4]. Numerous studies have demonstrated symptomatic relief and improvement in muscle function following occlusal splint therapy [4, 5]. However, the quantitative morphometric changes in TMJ spaces-especially in the context of condylar repositioning or joint space adaptation-have not been thoroughly documented. Advanced imaging techniques like Cone Beam Computed Tomography (CBCT) and Magnetic Resonance Imaging (MRI) provide three-dimensional, high-resolution assessment of both osseous and soft-tissue changes, offering valuable insight into post-therapeutic remodeling patterns [6, 7- 8]. Recent investigations, such as those by Jiang *et al.* [9, 10] have explored condylar dimensional and positional variations in patients with TMJ dysfunction, underscoring the importance of morphometric analysis. Yet, limited data exist that concurrently evaluate both CBCT and MRI-based metrics in the same cohort before and after splint therapy. Furthermore, factors like chewing side preference, unilateral masticatory dominance and lateral facial asymmetry-which potentially influence TMJ biomechanics-remain underrepresented in such radiological assessments [11, 12, 13, 14, 15, 16- 17]. In particular, chewing side preference and associated condylar activity have been linked to asymmetric joint loading, influencing disc position, joint space width and muscle volume [18, 19, 20-21]. Several authors have highlighted the role of unilateral function in the genesis of degenerative joint changes [22, 23] while others have advocated for occlusal therapy to reverse or mitigate these effects [24]. Despite these findings, longitudinal data capturing both soft tissue (disc-condyle relationship via MRI) and osseous (joint space and condylar position via CBCT) alterations remain sparse. Therefore, it is of interest to bridge the knowledge gap by quantitatively assessing the TMJ joint space changes pre- and post-occlusal splint therapy in 112 bruxer patients, utilizing both CBCT and MRI modalities.

## Materials and Methods:

A prospective observational study was meticulously conducted over a 12-month period to evaluate the therapeutic efficacy of occlusal splint therapy in individuals clinically diagnosed with bruxism accompanied by myofascial pain. This investigation aimed to capture both osseous and soft tissue alterations within the temporomandibular joint (TMJ) complex, employing advanced imaging modalities-Cone Beam Computed Tomography (CBCT) and Magnetic Resonance Imaging (MRI)-for structural and functional analysis. A total of 112 patients (56 males and 56 females) aged between 20 and 45 years were enrolled, all meeting strict inclusion criteria: confirmed clinical diagnosis of bruxism with associated myofascial pain, no prior TMJ therapy, and absence of systemic joint disorders such as autoimmune or inflammatory arthropathies. Patients with a history of TMJ surgery or trauma, those using muscle relaxants, antidepressants, or with contraindications to MRI were excluded to ensure diagnostic clarity and eliminate potential confounders. The CBCT scans were utilized to evaluate osseous componlents by measuring superior joint space (SJS), anterior joint space (AJS) and posterior joint space (PJS), while MRI scans assessed soft tissue parameters including articular disc displacement, joint effusion and joint capsule thickness. Data acquisition occurred at two critical time points: T1 (baseline, prior to initiation of therapy) and T2 (after six months of consistent nocturnal use of the occlusal splint). All participants were fitted with custom-fabricated hard acrylic maxillary stabilization splints, adjusted for bilateral balanced occlusion, and fine-tuned periodically to maintain neuromuscular equilibrium and vertical dimension stability.

For statistical evaluation, all quantitative variables were expressed as mean ± standard deviation. Paired t-tests were employed to compare pre- and post-treatment values of joint space measurements and MRI-derived soft tissue characteristics. In addition, chi-square tests were utilized for categorical variables such as the presence or absence of disc displacement and joint effusion. A p-value of <0.05 was considered statistically significant. Data were analyzed using SPSS software version 25.0, and results were further subjected to inter-observer reliability testing to ensure consistency in radiographic measurements. This rigorous design and analytical framework allowed for a comprehensive assessment of the structural and functional modifications associated with occlusal splint therapy in bruxism-related temporomandibular dysfunction.

## Results:

The demographic profile of the study participants revealed a mean age of 31.7 ± 6.4 years, indicating a predominance of young to early middle-aged adults. The gender distribution was equal, with 50% of the subjects being male and 50% female, ensuring a balanced representation across sexes for comparative analysis and minimizing gender-related confounding in the interpretation of therapeutic outcomes. The comparative analysis of CBCT-derived joint space parameters between baseline (T1) and post-therapy (T2) revealed statistically significant improvements across all measured dimensions following six months of nocturnal occlusal splint therapy. Specifically, the Superior Joint Space (SJS) exhibited a notable increase from a pre-therapy mean of 2.68 ± 0.43 mm to a post-therapy value of 3.12 ± 0.38 mm. This change was highly significant (p < 0.001), suggesting a vertical decompression effect in the superior compartment of the temporomandibular joint, likely attributable to the splint-induced condylar repositioning and reduction in myofascial load. Similarly, the Anterior Joint Space (AJS) demonstrated a mean increase from 2.12 ± 0.51 mm at T1 to 2.45 ± 0.44 mm at T2, with statistical significance (p < 0.001). This anterior repositioning may reflect an adaptive response of the condyle-disc complex toward a more physiologic alignment under stabilized occlusal conditions. The Posterior Joint Space (PJS) also increased significantly from 2.87 ± 0.39 mm to 3.23 ± 0.36 mm (p < 0.001), indicating a posteriorly directed shift of the condylar head, likely reducing posterior joint compression and correlating with subjective pain relief in many participants (Table 1 - see PDF).

The comparative evaluation of MRI parameters at baseline (T1) and after six months of occlusal splint therapy (T2) demonstrated substantial improvements in key soft tissue indicators of temporomandibular joint dysfunction. At T1, disc displacement was observed in 58 patients (51.8%), indicating a high prevalence of anterior or posterior disc dislocation within the study population. Following splint therapy, this number markedly reduced to 24 patients (21.4%), highlighting significant recapture or realignment of the articular disc. This suggests that prolonged stabilization and neuromuscular balance may contribute to disc repositioning and joint normalization. Joint effusion, a radiological marker of intra-articular inflammation, was initially detected in 37 patients (33.0%). Post-treatment MRI scans showed a dramatic decline, with only 9 patients (8.0%) exhibiting effusion, indicating a significant reduction in synovitis and inflammatory burden after therapy. Moreover, capsule thickening greater than 2 mm, which reflects chronic joint irritation or fibrotic changes, was present in 43 patients (38.4%) at baseline. This number decreased to 18 patients (16.1%) after six months of splint usage, reinforcing the potential of occlusal therapy in modulating chronic degenerative changes and improving soft tissue integrity within the TMJ (Table 2 - see PDF).

A comparative evaluation across gender subgroups revealed that both male and female participants exhibited significant improvements in joint space dimensions and disc positioning following occlusal splint therapy. However, no statistically significant difference was observed between the genders in terms of therapeutic response, as indicated by a p-value > 0.05. This suggests that the effectiveness of stabilization splint therapy is equally distributed across male and female patients, regardless of sex-based anatomical or neuromuscular variations. When analyzed by age, patients within the 20-30 years age group demonstrated a more rapid response in the normalization of joint space parameters during the early phases of therapy. This could be attributed to higher adaptive capacity and better tissue remodeling potential in younger individuals. Nevertheless, by the end of the six-month follow-up period, the final clinical and radiological outcomes were comparable between the younger (20-30 years) and older (31-45 years) age cohorts, indicating that age influenced the rate but not the extent of therapeutic improvement.

## Discussion:

Temporomandibular joint disorders (TMJD) represent a prevalent category of musculoskeletal dysfunction affecting the orofacial region, with an estimated 5% to 12% of the population experiencing symptoms severe enough to seek treatment [25]. Despite their widespread occurrence, the diagnostic and therapeutic approaches to TMJD remain heterogeneous, with considerable variability among dental practitioners in clinical decision-making and referral patterns [26]. Among the multifactorial etiologies associated with TMJD, bruxism-a parafunctional activity involving involuntary grinding or clenching of the teeth-has been consistently identified as a key contributor [27]. The relationship between bruxism and TMJ pathology, particularly in the context of biomechanical stress and joint overload, continues to be a subject of clinical investigation and debate [28]. The observed significant improvements in CBCT-derived joint space measurements following six months of nocturnal occlusal splint therapy affirm the therapeutic efficacy of this non-invasive modality in managing temporomandibular joint disorders (TMJD). The substantial increase in the superior joint space (SJS), anterior joint space (AJS) and posterior joint space (PJS) post-therapy suggests favorable condylar repositioning, improved intra-articular decompression and likely restoration of physiologic joint loading patterns. These findings resonate with previous studies by Musa *et al.*, who demonstrated both quantitative and qualitative improvements in condylar position and morphology using stabilization splint therapy assessed via CBCT [2, 3]. Their results showed a comparable trend of increased joint space and reduced articular surface stress, reinforcing the validity of the current study's outcomes. Likewise, Ok *et al.* reported glenoid fossa remodeling and condylar recentering as adaptive responses to long-term splint use, attributing such changes to modified neuromuscular control and improved occlusal stability [5]. The vertical gain in the superior joint space observed in our cohort is indicative of a superior condylar shift, consistent with the findings of Ikeda and Kawamura, who emphasized that stabilization splints support optimal condylar positioning by reducing anterior disc displacement and excessive joint compression. The posterior shift reflected by the increased PJS can be correlated with pain mitigation and muscle relaxation, as reported by Manrriquez *et al.* who found significant reductions in headache frequency and myofascial symptoms following splint therapy [14]. Moreover, Haralur *et al.* highlighted the importance of balanced occlusion and chewing side preference in mitigating asymmetric loading on the TMJ. Our gender-balanced cohort allowed for the control of gender-related anatomical and biomechanical variables, providing a more accurate representation of splint-induced morphological normalization [4]. The results also align with Hilgers *et al.* who emphasized the precision and reproducibility of linear joint measurements using CBCT, validating the reliability of joint space alterations as objective endpoints in TMJ therapy studies [7]. While the present study did not assess osseous remodeling per se, the significant spatial changes noted may reflect adaptive bone responses under the framework of Wolff's Law, wherein altered mechanical loading induces skeletal reconfiguration [24]. Future studies incorporating volumetric and remodeling metrics are encouraged to further delineate these structural changes.

In essence, the current findings not only reinforce the biomechanical rationale of occlusal splint therapy in TMJD management but also mirror the evidence base established by multiple prior investigations, underscoring its value as a conservative and reproducible treatment modality in clinical practice. The present study demonstrated significant improvements in key MRI parameters-disc displacement, joint effusion, and capsular thickening-after six months of occlusal splint therapy (OST), reaffirming its therapeutic role in temporomandibular joint disorders (TMD). These findings are consistent with and extend the scope of previously published data. The marked reduction in disc displacement from 51.8% to 21.4% following OST aligns with the concept of enhanced disc-condyle coordination post-therapy. Similar recapture effects were observed by Musa *et al.*, who reported morphological improvements in condylar symmetry and disc position in patients undergoing stabilization splint therapy, with or without mandibular asymmetry, using cone-beam CT imaging modalities [23]. Their findings underscore that neuromuscular rebalancing and condylar remodeling can facilitate anatomical repositioning, corroborating our MRI-based outcomes. Joint effusion, a hallmark of intra-articular inflammation, declined from 33.0% to 8.0% in our cohort, pointing to reduced synovitis. Ok *et al.* also reported glenoid fossa remodeling and decreased inflammatory signals in TMD-associated osteoarthritis patient's post-splint therapy. These findings mirror the biomechanical decompression offered by splint therapy, as emphasized by Manrriquez *et al.* that associated splint-induced muscle relaxation with reduced TMD-related headache comorbidities [14]. Furthermore, the observed reduction in capsular thickening (>2 mm) from 38.4% to 16.1% suggests a reversal of chronic irritation and fibrosis, supporting the regenerative capacity of long-term OST. This is in line with Wolff's Law, which underlines the adaptability of bone and soft tissue under controlled mechanical load-a principle validated in TMJ remodeling studies by Yang *et al.* where post-orthodontic-surgical intervention led to favorable condylar repositioning and adaptive osseous changes [16]. Although our study focused on soft tissue parameters, it parallels the conclusions of Shahidi *et al.* and Jiang *et al.* who demonstrated that morphological variations in the articular eminence and osseous structures correlate with dysfunction severity [8, 9]. These structural dynamics reinforce the relevance of early intervention with splint therapy to prevent progression toward degenerative joint disease. Collectively, these findings support the hypothesis that occlusal splint therapy not only alleviates symptoms but also induces substantial anatomic and physiological restoration in TMJ soft tissues. MRI remains a gold standard for monitoring these changes due to its superior soft-tissue contrast and non-invasive capabilities. The present study demonstrated that both male and female patients experienced significant improvements in joint space dimensions and disc positioning following occlusal splint therapy, with no statistically significant difference in therapeutic response between genders (p > 0.05). This aligns with prior research by Musa *et al.* who also found comparable condylar positional changes after stabilization splint therapy regardless of patient sex, suggesting that the biomechanical effects of splint therapy transcend sex-based anatomical or neuromuscular differences [3]. Similarly, Ok *et al.* [5] reported that glenoid fossa remodeling in TMJ osteoarthritis patients was not influenced by gender, supporting the notion that the therapeutic benefit of splints is uniform across sexes. From a clinical standpoint, this equivalence implies that stabilization splint therapy can be broadly applied without sex-specific modifications, reinforcing the clinical guidelines advocated by Asquini *et al.*, who emphasize standardization of manual and splint therapies across patient populations with TMJ disorders [10].

When stratified by age, our data showed that patients in the 20-30 years age group exhibited a faster initial normalization of joint space parameters during early therapy phases. This finding may be explained by greater tissue plasticity and enhanced adaptive remodeling capacity in younger individuals, consistent with Frost's Wolff's Law principle, which states that bone adapts structurally to mechanical stimuli more effectively during younger ages [2]. Moreover, the observation that the final outcomes at six months were similar between younger and older (31-45 years) cohorts resonates with the conclusions by Musa *et al.*, who noted that although younger patients might respond more rapidly, the overall extent of therapeutic improvement was not significantly age-dependent [3]. The temporary accelerated response in younger patients also correlates with findings by Ikeda and Kawamura, who reported that optimal condylar repositioning and adaptive remodeling in TMJ structures were more efficiently achieved in individuals with greater regenerative potential. Nonetheless, the convergence of treatment outcomes at later follow-ups suggests that stabilization splints effectively promote remodeling and symptomatic relief irrespective of age, albeit with different temporal dynamics. Overall, these findings underscore the importance of personalized patient education regarding expected timelines of improvement based on age, while reassuring both clinicians and patients of the universal benefit of splint therapy for TMJ disorders. This study underscores the importance of integrating advanced imaging in routine TMJ management for bruxers. Clinicians should consider pre- and post-therapy imaging as a part of protocol-based care to monitor treatment efficacy. On the managerial front, establishing a multidisciplinary approach incorporating radiology and prosthodontics enhances therapeutic outcomes and resource optimization in TMJ disorder clinics. The study's follow-up period was limited to six months, which may not capture long-term joint remodeling dynamics fully. Extended longitudinal studies incorporating larger, diverse cohorts and advanced imaging modalities are essential to optimize personalized occlusal splint protocols and enhance clinical decision-making for TMJ disorder management.

## Conclusion:

Occlusal splint therapy induces favorable morphometric changes in TMJ joint space in bruxer patients, as confirmed by CBCT and MRI evaluations. Data support the role of splint therapy not only in symptom relief but also in structural joint normalization. Both imaging modalities serve complementary roles in comprehensive TMJ diagnostics.
